# Design and *In-vitro *Evaluation of Sustained Release Floating Tablets of Metformin HCl Based on Effervescence and Swelling

**Published:** 2016

**Authors:** Faria Gias Senjoti, Syed Mahmood, Juliana Md Jaffri, Uttam Kumar Mandal

**Affiliations:** Department of Pharmaceutical Technology, Kulliyyah of Pharmacy, International IslamicUniversity Malaysia (IIUM), Kuantan, Malaysia.

**Keywords:** Metformin, sustained-release floating tablet, HPMC, PEO

## Abstract

An oral sustained-release floating tablet formulation of metformin HCl was designed and developed. Effervescence and swelling properties were attributed on the developed tablets by sodium bicarbonate and HPMC-PEO polymer combination, respectively. Tablet composition was optimized by response surface methodology (RSM). Seventeen (17) trial formulations were analyzed according to Box-Behnken design of experiment where polymer content of HPMC and PEO at 1: 4 ratio (A), amount of sodium bi-carbonate (B), and amount of SSG (C) were adopted as independent variables. Floating lag time in sec (Y_1_), cumulative percent drug released at 1 h (Y_2_) and 12 h (Y_3_) were chosen as response variables. Tablets from the optimized formulation were also stored at accelerated stability condition (40°C and 75% RH) for 3 months to assess their stability profile. RSM could efficiently optimize the tablet composition with excellent prediction ability. *In-vitro* drug release until 12 h, floating lag time, and duration of floating were dependent on the amount of three selected independent variables. Optimized tablets remained floating for more than 24 h with a floating lag time of less than 4 min. Based on best fitting method, optimized formulation was found to follow Korsmeyer-Peppas release kinetic. Accelerated stability study revealed that optimized formulation was stable for three months without any major changes in assay, dissolution profile, floating lag time and other physical properties.

## Introduction

Diabetes appears to be one of the most challenging diseases in terms of its devastating impact on global population. According to a recent report from world health statistics in 2012, every single person out of 10 suffers from this disease worldwide. Surprisingly, around 90% of all diabetic complications arise from Type II diabetes, alternatively known as non-insulin dependent diabetes mellitus (NIDDM). NIDDM results from excessive glucose present in the blood due to insulin deficiency with or without underutilization of insulin hormone produced by the body (1). Along with Type I diabetes, the prevalence of NIDDM is also increasing at epidemic proportion (2). Specific genetic profile in combination with sedentary life style in the form of high caloric intake and low exercise has been diagnosed as the cause of NIDDM (3). In addition to lifestyle modification, oral anti diabetic drugs and eventually insulin are required to maintain glycemic control for the majority of the patients (4).

Metformin HCl (MTH), most frequently prescribed by doctors in the treatment of NIDDM, belongs to biguanide class of antidiabetic agents (5). It has been very popular since its approval in the United States in December, 1994 and continued to be used as monotherapy or combination therapy with sulfonyl urea for the treatment and management of Type II diabetic patients (6). Its antidiabetic action is attributed to its ability to reduce glucose production from liver, reduce glucose uptake from gastro intestinal tract (GIT), and improve glucose utilization by skeletal muscle and adipocytes (7). It has been found from several studies that MTH is highly effective as well as safe in the treatment of Type II diabetes (8).

In spite of its favorable clinical response and lack of significant drawbacks, certain specific problems are observed in chronic therapy with MTH. Among all, the major problems are high dose (1.5-2.0 g/day), frequent dosing due to its shorter biological half-life (1.5-4.9 h), and low bioavailability (60%) (9). The marketed immediate-release products of MTH need to be administered 3 to 4 times daily (10). The rationality, therefore, exists for developing the drug as controlled-release formulation. As a consequence, many modified release medications of MTH are now available in the market; however the majority of them are formulated with matrix technology. These types of conventional formulations suit the drug candidates which are absorbed from the entire part of the GIT. Low bioavailability of MTH results from incomplete absorption of the drug as it is mainly absorbed from the stomach and the lower part of the GIT (11-12). In view of these problems, gastro-retentive formulation of MTH could be a better option than the existing immediate-release and conventional sustained-release counterpart. If the drug is designed to be released from its dosage form slowly over an extended period of time in the stomach, complete utilization of the drug might result in its enhanced bioavailability. This theory is supported by several studies which reported that the bioavailability of MTH improved when formulated as gastro-retentive drug delivery system (GRDDS) (13-16).

Various formulation approaches have been applied for the last few decades in developing GRDDS with a vast array of drugs. Some of the approaches include floating drug delivery systems, swelling and expanding systems, high density systems, ion-exchange resin systems, bio/muco-adhesive systems, raft forming systems, superporous hydrogel systems, magnetic systems, bioadhesive liposomal systems, and modified shape systems (17). Among these approaches floating drug delivery system has drawn huge interest as this system provides buoyancy action, which offers greater safety for clinical uses and is not affected by the peristaltic movement of the GIT (18). Low density (as compared to gastric fluid) achieved by this system inside the stomach fundamentally allows it to float in gastrointestinal fluid. Another important thing is that many floating dosage forms are currently doing well commercially and received positive feedback from the market (18-19). 

Several approaches of floating systems, as have been applied in the recent past, include hydrodynamically balanced system (HBS) based on hydrophilic polymers, gas generating or effervescence system, low density system, propylene tubes containing tablets or multi-units systems (20). Recently several more developments on this approach such as module assemblage technology (21), porous floating matrix system by the sublimation method (15), microsponge system (22), and fanicular cylindrical system (23), have produced encouraging *in-vitro* and *in-vivo* results on animals.

However, products showing good floating evidence during *in-vitro *and/or *in-vivo* experiments on animals, often exhibited unsatisfactory *in-vivo* performances in humans (20). In order to achieve gastric retention, a few formulation requirements should be complied. The dosage form must resist premature gastric emptying, *i.e*. have rapid buoyancy and must be resistant to peristaltic force inside the stomach and remain floating for a reasonably longer time (20). Additionally, the dosage form would act as a drug reservoir, releasing the drug slowly over a long time (20). To strictly follow these demands it is important to rationally select the appropriate polymers, in order to achieve the desirable floating behavior and strength. In this context, the selection of the matrix polymer is of great importance.

Recently, application of polyethylene oxide (PEO) has drawn huge interest as an alternative of hydroxyl propyl methyl cellulose (HPMC) in controlled-release formulation (24). PEO is a non-ionic linear homopolymer of ethylene oxide, synthesized by catalytic polymerization of ethylene oxide in the presence of a metallic catalyst system (25). It is available commercially with various molecular weights and viscosity grades which provide wide scope in selection of appropriate type of PEO depending on physicochemical properties of the targeted drugs (26). It has the advantage of being insensitive to biological pH with high gelation ability, non-toxic and biodegradable in nature (26). When in contact with water, it forms a viscous gel structure which slowly gets eroded due to its water solubility; both attributes help modulate drug delivery rate effectively. It can be used for the delivery of drugs having a wide range of solubility profile. Like any other hydrophilic polymers, PEO hydrogel releases water soluble and water insoluble drugs by diffusion and erosion mechanism respectively. Molecular weight and concentration of PEO, and solubility of the drug candidate are determinants, which affect the drug release from that hydrogel matrix. There are many reports on PEO used for gastro-retentive formulation (26). PEO hydrates very fast and swells in gastric fluid to a size that can be retained in the stomach even in fed stage (27). It has a slippery characteristic which promotes retention of the tablet in the stomach in the fed state while gastric content tries to pass through pyloric sphincters. However, PEO alone may not help to sustain drug release for longer duration. It rather necessitates the use of another polymer with higher gel strength to retain its matrix integrity. This is the reason why the use of other polymers such as HPMC along with PEO is required; stronger gel strength will result in desired drug release rate and floating duration in the stomach (26-30).

The objective of the present study was to fabricate, optimize and characterize sustained-release floating tablets of MTH based on the combination of swelling and effervescence mechanism.

## Experimental

*Materials*

MTH was purchased from Apollo Health Care Ltd., China. PEO (Polyox WSR 301) and HPMC (Methocel K100M premium DC grade) were generous gift samples from Colorcon Asia Pacific Pvt. Ltd, Singapore. Sodium starch glycolate (SSG), talc, magnesium stearate, sodium bicarbonate, polyvinyl pyrrolidone (PVP K30), isopropyl alcohol, dicalcium phosphate (DCP) were purchased from Fisher Scientific UK limited, UK. Other reagents utilized for the present study were of analytical grade. Active drug and excipients used for tablet formulation were received together with the certificates of analysis (COA) from the manufacturers. All necessary information on various physicochemical properties of the product was evident on the COA.

*Preformulation parameters*

Determination of various important preformulation attributes such as angle of repose, bulk density, tapped density, Carr’s index and Hausner ratio are essential to determine the flow property and compression property of powder/granules prior to tablet compression. The detailed procedures of these attributes are in the following sections:

*Bulk Density (BD)*

Pre-weighed powder or granule sample was sieved through 18 mesh screen, poured into a 100 mL graduated cylinder, and its volume was recorded. The same procedure was repeated three times, and the readings were averaged. Bulk density was calculated according to equation* 1* and expressed in gm/mL. 

equation (1) Bulk Density =Mass of powder Volume of packing

*Tapped density (TD)*

A pre-weighed powder sample or granule was put in a 100 ml graduated cylinder and fixed onto tap densitometer (JV 1000, Copley). The apparatus was run for a time until it achieved a constant volume (tapped volume). Tapped density was determined according to equation* 2* and expressed in gm/mL. 

equation (2) Tapped Density =Mass of powder Tapped volume of packing

*Compressibility Index (CI)*

CI is used to predict flowability of powder blend prior to compression. It was calculated according to equation 3:

equation (3) CI=Tapped density – Bulk density Tapped densityX 100

*Angle of repose*

Powder or granule flow property at preformulation stage can be determined by this simple and quick test. Cone forming method with fixed base was applied to determine the angle of repose. Powder sample or granule, devoid of any aggregation, was poured from a fixed height of about 10 cm to a fixed base (r) through a funnel supported by stand to form a symmetrical cone of powder mass. The height of the powder mass (h) was measured in triplicate to calculate the angle of repose (Ɵ) according to equation* 4*. 

equation (4) $$\theta =\tan^{-}\frac{h}{r}$$

*Drug-excipient compatibility studies*

Differential Scanning Calorimetry (DSC) study was performed to determine drug-excipient compatibility. Pure sample of drug and excipients individually and their physical mixtures as per formulation composition were run on DSC apparatus (Mettler Toledo DSC821e, Switzerland). A sample of approximately 10 mg was passed through 60-mesh sieve and loaded in the pierced DSC aluminium pan. The sample was scanned within a temperature range of 50 to 300˚C with a heating rate of 10˚C/min with an inert atmosphere of dry nitrogen. Obtained DSC thermograms were analyzed to confirm any drug-excipient compatibility (31). Apart from DSC, the pure drug and excipients, and their physical mixtures were analyzed by an IR spectrophotometer (Schimadzu IR–Prestige21) within a range of 4000-400 cm^-1^.

*Fabrication of MTH floating tablets*

Sustained-release floating tablets of MTH were fabricated by wet granulation method with the ingredients listed in Table 1. MTH and excipients excluding magnesium stearate and talc were passed through the sieve no.60. Accurately weighed quantities of MTH, HPMC, Polyox, NaHCO_3_, SSG and DCP were mixed geometrically for 10 min to ensure uniform mixing of all the components. Dry powder blends were subjected to granulation by PVPK30 solution in isopropyl alcohol (IPA). Granules were dried at 45-50˚C in a hot air oven (Universal oven UM 400/Memmert) to a moisture content of 2 to 3%. The dried granules were passed through a sieve no 18 and lubricated with magnesium stearate and talc for further 2 min. Eventually gastro-retentive MTH tablets were fabricated from the final blend by a 10-station Rotary tablet press machine (Mini press II/Remek, Karnavati Engineering Ltd., India) using 12 mm circular, biconcave punches. All the tablets were stored in airtight containers for further study. Uniform thickness (5.7–5.9 mm) and hardness (165-171 N) were maintained for all produced tablets. 

**Table 1:** Composition of sustained-release floating tablet of MTH.

**Ingredient**	**Amount (mg)**
Metformin HCl	500
Methocel K100M premium (HPMC)	25 to 50
Polyox WSR 301(PEO)	100 to 200
PVP K30	60
Sodium starch glycolate (SSG)	40 to 60
Sodium bicarbonate	40 to 60
Talcum powder	5
Magnesium stearate	5
Dicalcium phosphate	qs to 950

qs, quantity sufficient.

*Floating lag time and floating duration*

Method reported by Tadros was utilized to measure floating lag time and duration of floating for the developed tablets (32). One tablet was put in a 200 mL beaker containing 0.1 N HCl at 37˚C. Floating lag time was determined as the time taken for that tablet to reach to the surface, whereas duration of floating was measured visually as long as it maintained its floating in 0.1 N HCl. The test was performed in three replicates, and the result was averaged.

*In-vitro dissolution study*

USP type I (basket method) dissolution testing apparatus (Copley/DIS 8000) was used to measure *in-vitro* drug release profile of the developed sustained-release floating tablets (27). Each basket was filled with 900 mL of 0.1 N HCl as the dissolution medium maintained at a temperature of 37 ± 0.5˚C. The entire test was conducted at 100 RPM. Samples of 5 mL were withdrawn from an individual basket at predetermined time intervals (1, 2, 4, 6, 8, 10 and 12 h) and equal volume of a freshly prepared dissolution medium was replaced immediately. After filtration (150 mm whatman filter paper) and appropriate dilution with dissolution medium, the test samples were analysed by UV-Spectrophotometer (Hitachi/UV-1900) at a wavelength of 232 nm. LOQ (lower limit of quantification) of UV-visible spectrophotometric method was found to be 1 μg/ml with a linear relationship between peak areas and MTH concentrations in the range of 1 to 12 μg/mL (r^2^ = 0.9989, equation: y = 0.0851x + 0.0015). Inter-day precision values for the developed UV method also were found to be within the acceptable limit of ICH method validation guidelines.

*Formulation optimization by Response Surface Methodology*

Response surface methodology (RSM) with 3 levels, 3 factors Box-Behnken design was applied to optimize the proposed formulation. Box-Behnken design was preferred as choice of experimental design considering its requirement of fewer numbers of experimental runs for three or four independent variables than full factorial design (33). Concentration of hydrophilic swelling polymers, swelling enhancer and effervescent component have significant influence on floating lag time and drug release of a GRDDS as reported by several researchers (29, 34). Therefore, the total amount of HPMC and PEO in the ratio of 1: 4 (A), SSG (B) and NaHCO_3_ (C) were selected as independent variables considering their overall effects on the response variables such as floating lag time (Y_1_), percentage drug released at 2 h (Y_2_), and 12 h (Y_3_) (Table 2).

**Table 2:** Variables of Box-Behnken design for fabrication of sustained-release floating tablets of MTH.

**Independent variables**		**Levels of factors**		
		**+1 (High)**	**0 (Medium)**	**-1 (Low)**
A	HPMC: PEO (1:4)	50:200	37.5:150	25:100
B	NaHCO_3_	60	50	40
C	SSG	60	50	40
**Dependent variables**				
Y_1_ = Floating lag time (sec) Y_2_ = Cumulative % drug release at 2 h Y_3_ = Cumulative % drug release at 12 h				

*Experimental trials originated from Box-Behnken design*

The complete Box-Behnken experimental design consisted of 12 factor points and 5 replications at the center points; altogether, 17 experimental runs that are illustrated in Table 3. Other composition and manufacturing conditions were kept constant for all experimental runs.

**Table 3:** Three levels three factors Box-Behnken design for sustained-release floating tablets of MTH.

**Formulation code**	**Independent variables**			**Dependent variables**		
	**A**	**B**	**C**	**Y**_1_	**Y**_2_	**Y**_3_
F1	0	+1	-1	160	37.21	97.56
F2	0	0	0	240	39.30	93.82
F3	0	0	0	230	40.38	95.55
F4	0	0	0	240	42.36	94.04
F5	+1	0	-1	200	35.88	90.08
F6	0	-1	-1	225	36.24	95.23
F7	-1	0	-1	180	43.12	95.30
F8	+1	0	+1	190	32.66	90.15
F9	0	0	0	220	38.34	95.03
F10	+1	-1	0	230	35.23	93.18
F11	-1	-1	0	210	40.63	97.00
F12	-1	+1	0	140	41.24	94.13
F13	0	-1	+1	215	40.81	95.82
F14	+1	+1	0	190	33.28	91.82
F15	-1	0	+1	200	39.47	94.25
F16	0	+1	+1	145	36.34	93.38
F17	0	0	0	250	40.86	93.04

*Data analysis and RSM model validation*

RSM option of Design expert® software (version 8.0.7.1, stat-Ease Inc., Minneapolis, MN) was employed to perform optimization of developed sustained-release floating tablet of MTH composition. Multiple linear regression analysis (MLRA) option of the software allows to find statistically significant most appropriate linear and quadratic polynomial models containing individual (A, B, and C), interaction (AB, BC, and AC) and quadratic (A^2^, B^2^, and C^2^) terms. Mathematical forms of proposed linear and quadratic models are described by equation *5* *and *equation *6* respectively.

*Y *= α_0_ + α_1_*A *+ α_2_*B *+ α_3_*C                     *(*5*)

*Y *= α_0_ + α_1_*A *+ α_2_*B *+ α_3_*C *+ α_12_*AB*+ α_13_*AC*+ α_23_*BC *+ α_11_*A*^2^ + α_22_B^2^ + α_33_C^2^                     (*6*)

Where *Y* is the model predicted response value consorted with all independent variables; α_0 _is the constant value which is calculated from the arithmetic mean of experimentally obtained response variables of 17 experimental runs; α_1_, α_2_, α_3 _are linear coefficients calculated from the experimentally obtained response values of *Y*; α_12_, α_13_, α_23 _are interaction coefficients of all three independent variables when considered any two of them at a time, whereas α_11_, α_22_, α_33_ are quadratic coefficients of those independent variables (35). Two dimensional (2-D) contour plots were generated for individual response variable in order to evaluate the effects of individual independent variables on individual response variable. Developed model was further validated based on experimental versus predicted values and their corresponding residual plot. Finally, the best formulation was selected from 17 trial formulations based on achieving optimum values set for the response variables. 

*Characterization of optimized gastro- retentive tablets*

Tablets from optimized formulation were characterized for their routinely performed quality control (QC) parameters like uniformity of weight, thickness, hardness, friability, drug content, and percent of moisture content. Additionally, they were also tested for floating lag time, floating duration, and *in-vitro* drug release behavior.

*Uniformity of weight*

It is very important to maintain weight uniformity of tablets within a batch as well as among batches of the same weight. This test represents that all the tablets in a single batch are of same potency, within acceptable limits. Twenty tablets were selected at random, weighed individually and the average weight of the tablets was calculated from the total weight. Weight variation was calculated by comparing weight of individual tablet with the average weight (36). 

*Hardness and thickness*

Determination of hardness is required to assess the resistivity of tablets against mechanical shocks during their handling in manufacturing, packaging, and shipping operations. Tablet hardness is measured as compression force to break a tablet while applied diametrically. Tablet thickness, on the other hand, affects its packaging operation. Ten tablets were selected at random and measured for hardness and thickness simultaneously by using the Pharma test (PTB/411) hardness tester (36). The average thickness with standard deviation was reported.

*Friability*

If the uncoated tablets are not strong enough to withstand mechanical shock subjected to coating, packing or transporting, they may undergo chipping, capping or breaking problem. This problem may arise if the tablet ingredients are not cohesive enough to bind them together. Friability test is performed just to assess that cohesive force.

Ten tablets were weighed, put into Roche friabilator (Sotax/CH 4123) and run for 4 min at 25 rpm. During each revolution the tablets were subjected to fall from a height of 6 inches. After completion tablets were cleaned to make them free from any adhered dust and reweighed. Friability was calculated by equation* 7. *The test was acceptable under the pharmacopoeal limit of less than 1% weight loss (36).

equation (7) $$\mathrm{\% friability}=\frac{W_{0}-W}{W_{0}}\mathrm{X 100}$$

Where, W_o _is initial weight of tablet and 

 W is the final weight of tablet after the friability test

*Drug content*

To ensure uniform distribution of MTH within the developed tablets, drug content (assay) was verified. Twenty tablets were randomly selected, weighed and powdered with mortar and pestle. The quantity of powder equivalent to 100 mg of MTH was transferred carefully into a 100 mL volumetric flask and dissolved the drug within the powder with 0.1N HCl. Then the solution mixture was filtered and after an appropriate dilution, the filtrate was spectrophotometrically measured at 232 nm against 0.1 N HCl as blank. Drug content was estimated from the standard curve as mentioned in *section 2.6*. 

*Moisture Content*

Moisture analyzer (Halogen moisture analyzer/HB43) was used to determine the moisture content of the fabricated tablets. Ten tablets were crushed, approximately 5 g powder sample was loaded in the pan and apparatus was run at 105ºC for 10 min. The result was recorded in triplicate.

*Cumulative % swelling*

The swelling behavior of the tablet was measured by equilibrium weight gain method (15). It was carried out in a dissolution apparatus (Copley/DIS 8000) without any stirring of the dissolution medium. Pre-weighed tablets (W_0_) were placed in the basket containing 900 ml 0.1N HCl dissolution medium maintained at 37 ± 0.5˚C. The swollen tablets were taken out from the dissolution basket at predetermined time intervals t (0, 0.25, 0.5, 1, 2, 4, 6, 8 and 12 h). At each time point (t), tablets were taken out of the dissolution basket; excess water adhered to the swollen surface was soaked with tissue paper immediately and weighed on the analytical balance (W_t_) (Mettler Toledo, AB125-S/FACT). The experiment was performed in three replicates. Cumulative % swelling was calculated by using equation* 8*.

equation (8) $$\mathrm{Cumulative \% swelling}=\frac{W_{t}-W_{0}}{W_{o}}X100$$

*Drug release kinetics*

The *in-vitro* drug release data from tablets of optimized formulation were applied to zero-order (37), first-order (38), Higuchi (39), Hixon-Crowell (40), and Weibull (41), and Korsemeyer and Peppas model (42) model to find out the drug release kinetic. 

*Scanning electron microscopy (SEM)*

SEM study provides valuable information to confirm *in-vitro* drug release and floating mechanism of a GRDDS. During dissolution study, samples were taken in the different time interval (0, 2, 4 and 8 h) and blotted to remove excess water. Then they were dried in an oven (Universal Oven (UM 400/Memmert)). After that the samples were gold coated with Cool Sputter Coater (Leica EM SCD005), and viewed under Carl Zeiss scanning electron microscope (EVO series) at an accelerating voltage of 8 to 10 Kv.

*Accelerated stability study*

The tablets from optimized formulation were tested for stability under accelerated storage condition (40˚C and 75%RH) for 3 months in accordance to ICH guidelines. The sampling was done at 0, 1, 2 and 3 months. Each time samples were evaluated for appearance, color, odor, size, hardness, friability, moisture content, drug content, *in-vitro* dissolution profile, and *in-vitro* buoyancy parameters such as floating lag time and duration of floating. All Results were compared against optimized formulation of 0 month as the reference.

## Results

*Preformulation studies*

*Pre-compression parameters of granules*

Results of pre-compression parameters of granule/powder blends are illustrated in Table 4. Estimated values of angle of repose (< 35˚), Carr’s index (< 15), and Hausner ratio (< 1.25) indicate that flow properties of powder blend were fair to facilitate smooth operation of tableting (36). 

**Table 4:** Results of pre-compression parameters of powder blend for MTH floating tablets

	**Angle of repose** **(Degree)**	**Bulk density** **(gm/cc)**	**Tapped density** **(gm/cc)**	**Carr’s Index**	**Hausner ratio**
Run 1	31.1	0.426	0.448	4.91	1.05
Run 2	31.6	0.423	0.447	6.00	1.06
Run 3	32.1	0.425	0.450	4.92	10.5
Average value ± SD	31.6 ± 0.5	0.424 ± 0.001	0.448 ± 0.001	5.27 ± 0.60	1.05 ± 0.01

*Drug-excipient compatibility studies*

**Figure 1.**
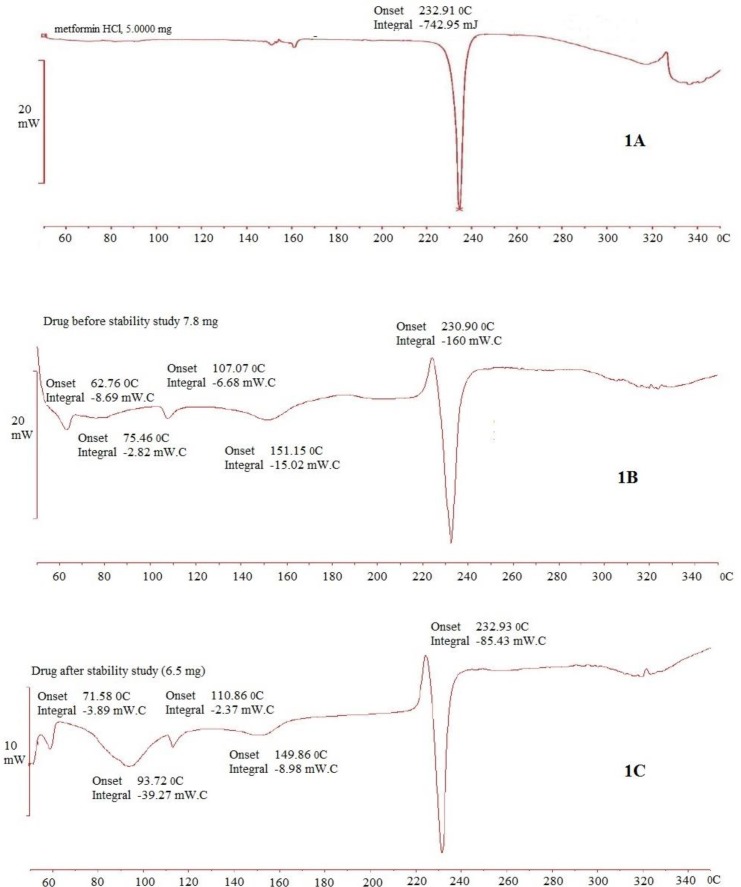
DSC thermograms of MTH (1A), its mixture with other excipients before (1B) and after (1C) accelerated stability studies

**Figure 2:**
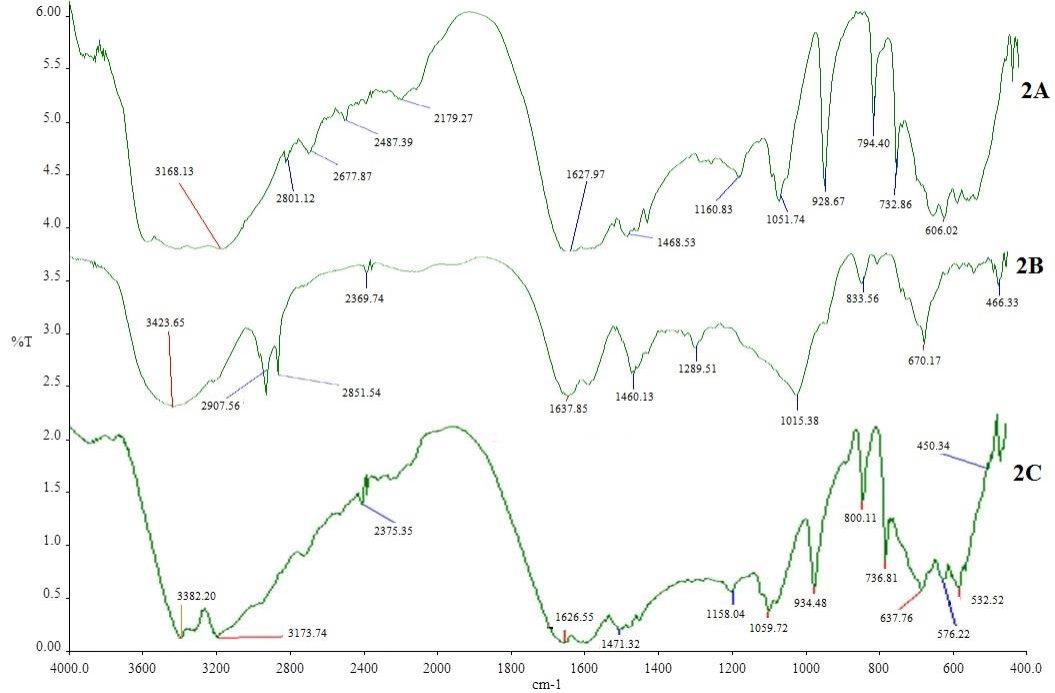
FTIR spectrum of MTH (2A), its mixture with other excipients before (2B) and after (2C) accelerated stability studies

The DSC thermograms of pure MTH, its physical mixture with other excipients (before and after of accelerated stability studies) are shown in Figure 1. Thermogram 1A exhibits a sharp endothermic peak at 232.91°C which corresponds to melting point of MTH. When MTH was mixed with other excipients, thermog 1B and 1C still retained drug peak, which is the indication of MTH compatibility with other excipients used for the proposed formulation composition. This was further confirmed by comparing the DSC thermograms of individual pure excipient against the physical mixture before and after the stability study (data not shown). Similarly, FTIR spectrum of pure MTH (2A) in Figure 2 exhibits entire characteristic peak N-H stretching, C = N stretching, C-N stretching, and C-H bending at 3397.96 cm^-1^, 1627.97 cm^-1^, 1051.74 cm^-1^, and 1468.53 cm^-1 ^respectively when compared with reported reference spectrum of the drug (43). These distinctive drug peaks were present in FTIR spectrum of physical mixture of the drug with other excipients before the accelerated stability study (Figure 2B) and the optimized tablet after the accelerated stability study (Figure 2C) as well. From the DSC and the FTIR studies, it can be concluded that MTH is highly compatible with other excipients used in the formulation. 

*Optimization of MTH sustained-release floating tablets by RSM*

*Selection of independent variables for Box-Behnken design*

The primary objective of this research work was to design a sustained-release floating tablet of MTH based on the combination of swelling and effervescence mechanism that would result in minimum floating lag time, remain buoyant for extended period of time and sustain the drug release for 12 h. Several formulation variables were considered towards the fulfillment of that objective. PEO swells rapidly, but is unable to retain its swelling matrix for a longer period (27). Its rapid swelling helps to decrease the effective density of the gel matrix below 1gm/mL, which is important for floating of the system. HPMC, on the other hand, provides integrity in the gel matrix when combined with PEO and helps to retard the drug release of a highly water soluble drug like MTH (27). This is the reason why the combination of HPMC and PEO at 1: 4 (A) was selected for this study. Sodium bicarbonate (B) acts as a gas forming agent, whereas SSG (C) as swelling enhancer. The preliminary study of the developed tablets without sodium bicarbonate resulted longer floating lag time (data not shown). Therefore, considering the importance of effervescence along with polymer swelling, we included sodium bicarbonate as a gas-generating agent within the formulation. The level of the independent variables was selected based on the initial experiments followed by their observations (data not shown).

*Responses/results of dependent variables for all experimental trials*

Various combinations of three independent variables (A, B, and C) according to Box-Behnken design generated 17 experimental trials, including five centre points (Table 2 and Table 3). All trial formulations were fabricated and analyzed for floating lag time in sec (Y_1_), percentage drug released at 2 h (Y_2_), and 12 h (Y_3_) in order to optimize the formulation composition by RSM. Results obtained for all trials are given in Table 3. Differences in drug release profile are clearly visible among the various trial runs which were good for RSM optimization exercise.

*Mathematical modeling*

Composition of sustained-release floating tablets of MTH was optimized by Design expert® software. Obtained response values of individual trial formulations were fitted in appropriate option of the software in order to find the best-fitted model. After due consideration, the best-fitted model for Y_1_ and Y_3_ were found to be quadratic, whereas for Y_2_ it was linear. Multiple linear regression analysis (MLRA) was applied to find out the mathematical relation among independent variables with a particular response variable. The obtained results of MLRA analysis to find out the quantitative effects of independent variables on three response variables are provided in equation* 9* to 11.

Y1=+236+10A-30.63B-1.87C+7.50AB-7.50AC-1.25BC-18.62A2-24.87B2-24.88C2                     

Equation (9)

Y2 = + 38.43-3.42 A-0.61B-0.40C                     Equation (10)

Y3=+94.30-1.93A-0.54B-0.57C+ 0.38AB+0.28AC-1.19BC-1.19A2+1.39B2-0.19C2                    Equation (11) 

Analysis of variance (ANOVA) was the selected statistical test to ensure that the developed model was statistically significant (Table 5). Using 5% significance level, a model was considered significant if the *p* value (significance probability value) is less than 0.05 (35). In Table 5, individual models, including several important terms (marked in bold) were found to be less than 0.05. Nature of interaction between independent variables to the response variables is qualitatively diagnosed by respective sign of the coefficient representing individual independent variables and their combination in the equation; a positive sign indicates a synergistic effect while an antagonistic effect is represented by negative one. From the above equations 9 to equation 11, it is evident that independent variables (first order) have a negative effect for all three responses except A in Y_1_. On the other hand, higher-order terms except *B*^2^ in equation 11, have negative effects on all response veriables. 

**Table 5:** Analysis of Variance (ANOVA) of all three response variables as per Box-Behnken design.

	**Y**_1_		**Y**_2_		**Y**_3_	
**Source**	**F-Value**	**P-Value**	**F-Value**	**P-Value**	**F-Value**	**P-Value**
Model	10.51	0.0026	7.52	0.0036	5.71	0.0158
A	4.66	0.0677	21.61	0.0005	25.39	0.0015
B	43.72	0.0003	0.67	0.4266	2.00	0.1999
C	0.16	0.6977	0.29	0.6000	2.22	0.1797
AB	1.31	0.2898	--	--	0.49	0.5086
AC	1.31	0.2898	--	--	0.27	0.6214
BC	0.036	0.8541	--	--	4.84	0.0637
A^2^	8.51	0.0224	--	--	9.85	0.0164
B^2^	15.18	0.0059	--	--	6.97	0.0334
C^2^	15.18	0.0059	--	--	0.13	0.7257

*Floating lag time (Y*_1_ *)*

Any floating dosage form should initiate floating immediately or after a minimum lag period in order to bypass the peristaltic movement and escape out from the stomach to the small intestine (19). Various formulation variables such as effects of concentration of swelling polymer, swelling enhancer, gas generating agent influenced the floating behaviour of the present study. The floating tablets were composed of sodium bicarbonate as gas forming agent and combination of PEO and HPMC as swelling matrix. Upon contact with the acidic medium, *i.e*. 0.1 N HCl, the fluid permeates into the matrix and initiates effervescence reaction. Liberated CO_2_ is entrapped within the polymeric network. Consequently, polymer matrix swells rapidly and the swollen tablet achieves a required density which initiates it to float, reaches on the surface and remains buoyant for a long time as long as it maintains the required buoyancy. 

**Figure 3:**
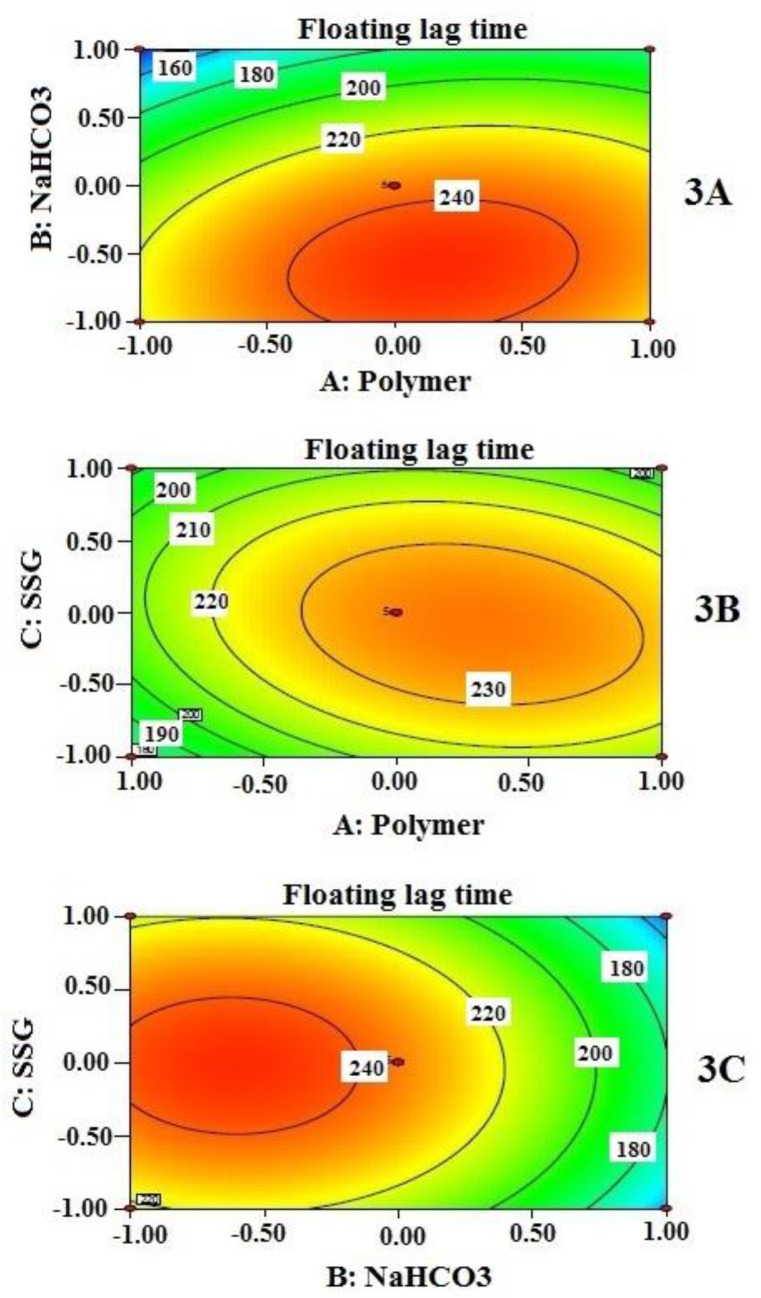
Contour plots which show the influence of independent variables (3A, 3B, and 3C) on floating lag time in sec (Y_1_). Two variables are considered at a time while third one remains constant

Effects of independent variables on floating lag time (Y_1_) are presented by 2-D contour plots in Figure 3. Floating lag time was found to be inversely related, not exactly proportional to the concentration of sodium bicarbonate when other two independent variables remained constant (Figure 3 (3A and 3C)). However, from contour plots, it is evident that sodium bicarbonate significantly influenced floating lag time when its amount is beyond 50 mg per tablet only; 60 mg produced the lowest value of floating lag time. This value may be considered as a requirement for minimum effervescence in order to equilibrate gravitational force with buoyancy force exerted on the tablet while floating. The effect of SSG (C) and polymer combination (A) on floating lag time, at a fixed amount of sodium bicarbonate (B), is also evident in Figure 3B. As it is clearly noticeable that the lower level of A and the entire range of C provide significant changes on floating lag time. 

*Cumulative % drug released at 2 h (Y*_2_ *)*

**Figure 4:**
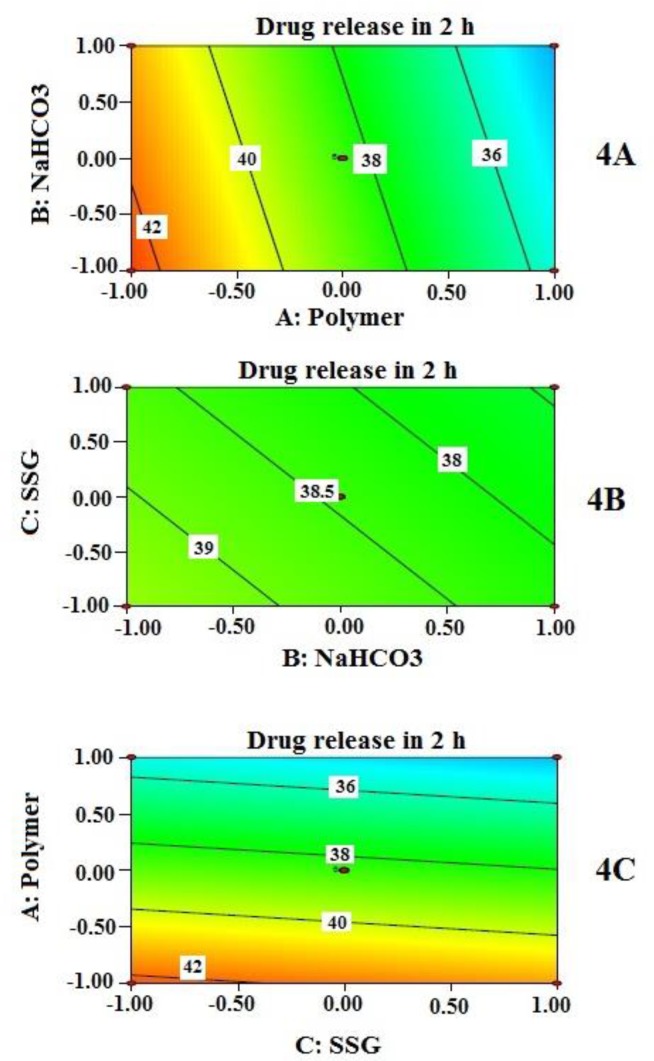
**.** Contour plots which show the influence of independent variables (4A, 4B, and 4C) on cumulative % drug release at 2 h (Y2). Two variables are considered at a time while third One remains constant

Effects of independent variables on % drug released at 2 h (Y_2_) are presented by 2-D contour plots (Figure 4). Cumulative % drug released at 2 h (Y_2_) was best described by a linear model instead of the quadratic model. This explains why the contribution of independent variables on Y_2_ was quite straight forward as detected by the three contour plots (Figure 4). However, the drug release is not sufficiently controlled unless all the independent variables reached a certain amount. Again, contribution of sodium bicarbonate to drug release appears to be negligible unless it reached a certain concentration as evident from the contour plots in Figure 4 (4A and 4B). At higher concentration of NaHCO_3_, it may have increased porosity followed by rapid hydration, which might influence the drug release. Similar finding was reported by Sungthongjeen *et al.* for floating multilayer coated tablet of theophylline with NaHCO_3_ as gas forming agent (34). The high value of drug release at the initial period can be further explained by high water solubility of MTH. The rapid swelling and hydrophilic nature of PEO in the presence of SSG even increase the porosity of the matrix which helps in rapid contact between drug (located near the periphery) and water. This might allow MTH that is located at the outer surface of the tablets to get released quickly. 

*Cumulative % drug released at 12 h (Y*_3_ *)*

**Figure 5:**
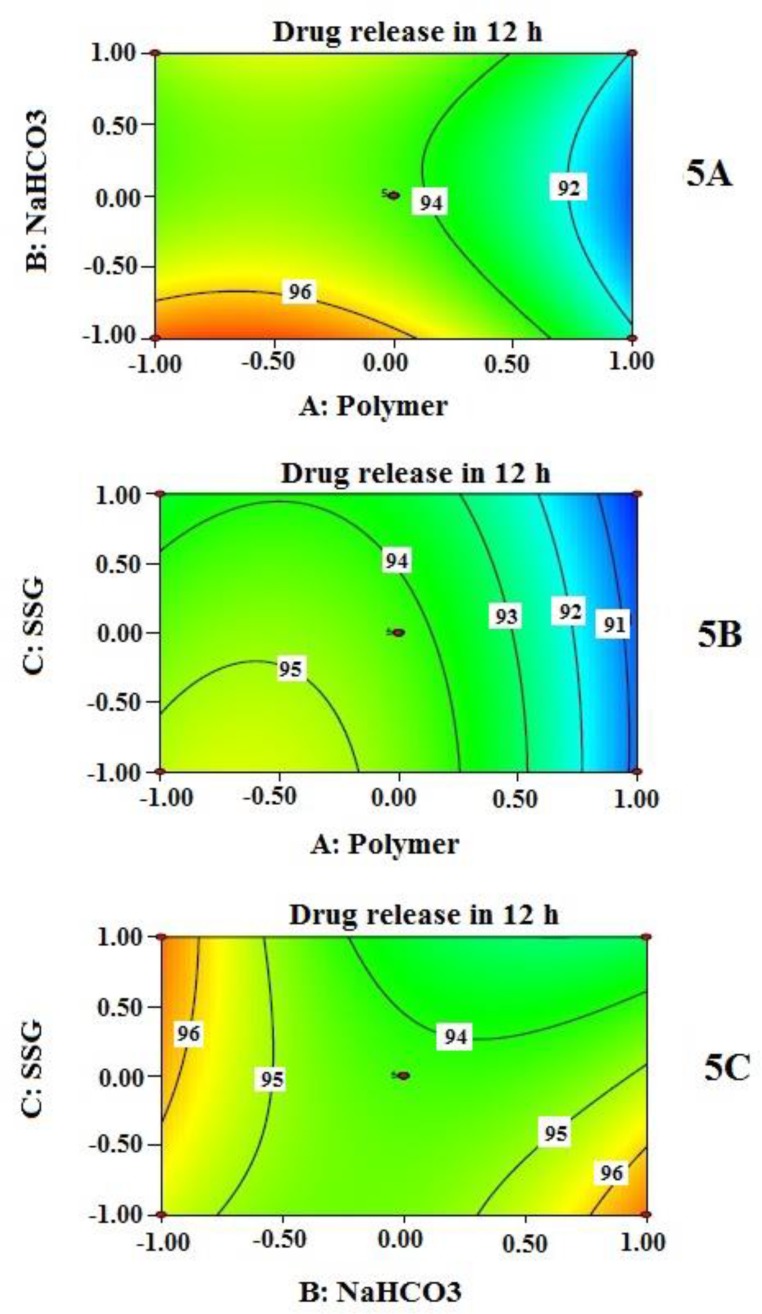
Contour plots which show the influence of independent variables (5A, 5B, and

Effects of independent variables on % drug released at 12 h (Y_3_) are presented by 2-D contour plots shown in Figure 5. As observed in contour plots, the amount of polymer combinations (A) influences the extent of drug release at 12 h; as the amount is increased, the drug release is decreased and vice versa. On contact with dissolution medium, very hydrophilic and the swelling nature of HPMC and PEO within the tablet initiate their transformation to a gel-like structure. This gel formation depends on the type of polymers, and their concentration used in the formulation. Viscous nature and thickness of gel structure control the drug release. This finding is also supported by many other researchers (15, 34). Swelling enhancer helps increase the rate of swelling of hydrophilic polymer. At a higher amount of the swelling enhancer SSG (when NaHCO3 remained fixed), % cumulative drug release decreases along with the increase of polymer combination (Figure 5 (5B)). This may be explained by the rapid increase of effective path length of drug diffusion. However, at the fixed amount of polymer (A), the contribution of NaHCO_3_ (B) and SSG (C) resulted in an interesting finding (Figure 5C); a combination of low value of B and high value of C produces similar drug release behavior at 12 h when the values are interchanged (high value of B and low value of C). The overall effect of Y_3_ is highly influenced by the amount of polymer combinations; the contribution of NaHCO_3_ (B) and SSG (C) are not that much predominant.

5C) on cumulative % drug release at 12 h (Y_3_). Two variables are considered at a time while the

third one remains constant.

*Internal validation of the model*

The theoretical values of Y_1_, Y_2 _and Y_3_ of the all formulation trials as per Box-Behnken experimental design were obtained by substituting their corresponding values of A, B and C in the respective equations (equation 9 to 11) generated by Design Expert software®. The theoretical (predicted) values and the observed values were in the reasonable good agreement as confirmed by the results obtained from the optimized formulation (Table 6), which proved the validity of the developed RSM model.

**Table 6:** Predicted and observed values of the dependable variables from the optimized formulation based on the developed best-fitted models and experimental results, respectively

**Dependent variable**	**Predicted**	**Observed**
Y_1_	179	190
Y_2_	34.40	33.28
Y_3_	92.41	91.82

*Selection of the optimized formulation*

Point prediction option of the software was utilized to select the optimized formulation out of 17 trial formulations prepared according to Box-Behnken experimental design. The optimized composition of present formulation was selected at the minimum values of Y_1_ and Y_2_ whereas the maximum value of Y_3_. After thorough evaluation by the software, it was found that formulation F14 with the polymer combination of HPMC and PEO at 1: 4 ratio (A) 250 mg, NaHCO_3_ (B) 60 mg, and SSG (C) 50 mg fulfilled the criteria of an optimum formulation. The optimized formulation produced floating lag time (Y_1_) 190 sec, 33.28% drug release at 2 h (Y_2_), and 91.82% drug release at 12 h (Y_3_).

*IPQC parameters of optimized tablet formulation*

Evaluation of IPQC parameter is very important step for product acceptance. Optimized sustained-released floating tablets of MTH were evaluated for its IPQC parameter and found to comply with acceptance criteria as shown in Table 7.

**Table 7:** IPQC parameters of optimized MTH sustained-released floating tablets.

**IPQC parameter**	**Inference**	**Acceptance Criteria**
General appearance	Colour: white to off-white; Shape: Oblong;	-
Weight variation (n=20)	-0.32 to + 0.62 %	± 5%
Tablet thickness (n=10)	5.60 ± 0.03 mm	
Hardness (n=5)	165 to 171 N	-
Friability (n=10)	0.11%	<1%
Moisture Content (n = 10)	2.03%	-
Floating behavior (n = 6)	Floating lag time: 3.46 ± 0.05 Min Floating duration: >24 h	-
Drug Content ( n = 20)	98 %	95-105%

*Drug release kinetic*

Table 8 summarizes *r*^2^ values and other related parameters of different mathematical models applied in order to confirm drug release kinetics. Optimized formulation did not follow zero order release kinetic at all. Based on the criteria of best-fitting method, optimized formulation was found to follow Korsmeyer-Peppas release kinetics (Table 8). To explore it in detail, Korsmeyer-Peppas model was studied further. Korsmeyer and his group proposed the following equation 12 to describe the drug release behavior from a polymeric drug delivery system (42) 

equation (12) $$\frac{M_{t}}{M_{\alpha }}=bt^{n}$$

Where, *M*_t_ is the amount of the drug released at time *t*, *M*_α_ is the total amount of drug present in the delivery device, *b *is the constant which represents structural and geometric configuration of the delivery device and *n *is the release exponent which indicates the drug release mechanism. Most of the times, drug release mechanism from a delivery device composed of hydrophilic polymeric excipients can be described by diffusion (Fickian diffusion). However, during the process of diffusion, the polymer network undergoes relaxation, which can influence overall drug release rate. This simultaneous drug release mechanism of diffusion and relaxation can be termed as non-Fickian or anomalous diffusion. This is further influenced by type (viscosity grade) and concentration and swelling property of the polymer. For a delivery system with fixed geometry, the value of n is found to be 0.5 in case of Fickian diffusion and above 0.5, but below 1.0 in case of non-Fickian diffusion. Apart from those two mechanisms, the third type of diffusion mechanism named as Case II diffusion is also frequently observed where the value of n is exactly 1.0 (42). For the present investigation, the value of n was found to be 0.548 which indicates non-fickian type of drug diffusion.

**Table 8:** Summary of mathematical modeling of release profile of an optimized formulation

**Model**	**Parameters**
Zero order	k_0_ = 8.665, R^2^ = 0.725, AIC = 50.477
First order	k_1_ = 0.177, R^2^ = 0.965, AIC = 36.147
Hixson-crowell	k_HC_ = 0.048, R^2^ = 0.940, AIC = 39.818
Higuchi	k_H_ = 25.700, R^2^ = 0.994, AIC = 24.127
Korsmeyer peppas	k_KP_ = 23.314, n = 0.548, R^2^ = 0.999, AIC = 15.536
Weibull	α = 147.810, β = 2.037, T_i_= -5.424, R^2 ^= 0.997, AIC = 22.020

k_0, _zero-order release constant; R^2^, coefficient of determination; AIC, Akaike information criterion; k_1, _first-order release constant; k_HC, _release constant in Hixson–Crowell model; k_H, _Higuchi release constant; k_KP, _release constant incorporating structural and geometric characteristics of the drug-dosage form obtained from Korsmeyer peppas model; n, diffusional exponent indicating the drug-release mechanism; α, scale parameter which defines the time scale of the process; β, shape parameter which characterizes the curve as either exponential (β = 1; case 1), sigmoid, S-shaped, with upward curvature followed by a turning point (β > 1; case 2), or parabolic, with a higher initial slope and after that consistent with the exponential (β < 1; case 3); T_i_: location parameter which represents the lag time before the onset of the dissolution process. 

*Cumulative % swelling*

**Figure 6:**
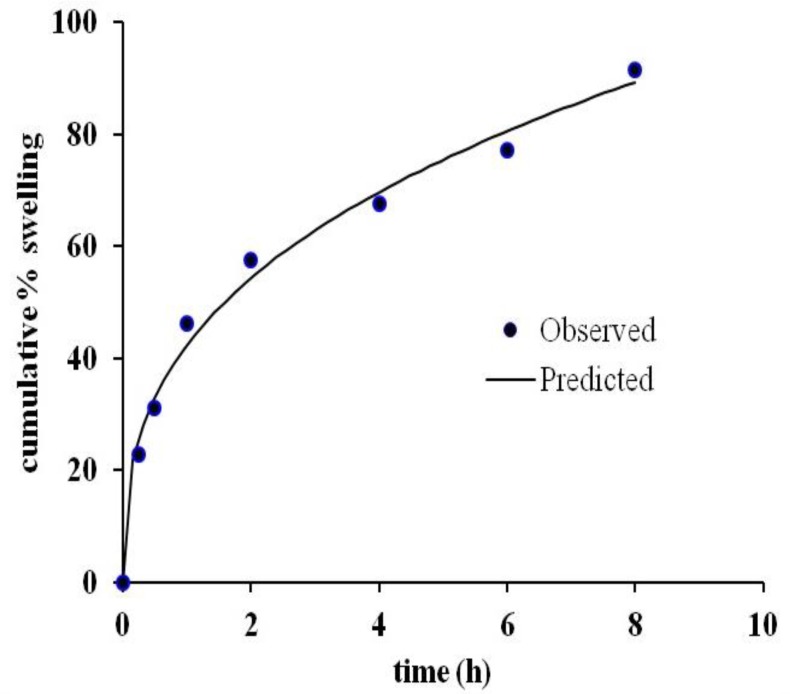
Observed and predicted cumulative % swelling according to Koresmeyer-peppas kinetic model.

Swelling study was performed on optimized sustained-release floating tablets. Interestingly, a good correlation (r^2^ = 0.9910) was found between observed and predicted values of cumulative % of swelling when fitted in Korsmeyer peppas kinetics model (Figure 6) which agrees also with drug release kinetics descried earlier. The rapid swelling nature of HPMC and PEO in the presence of SSG allow them to form a viscous gel quickly with an increase of effective path length of diffusion, which helps control the drug release rate. On the other hand, because of swelling, effective density of the tablet decreases, and it helps the tablet to remain floating as long as the system can maintain the required density for floating. Quick rate of swelling helps minimize floating lag time. However, the rate of swelling is not uniform throughout the study, it increased initially rapidly and then the rate decreased. The same finding has also been reported by Oh *et al.* (2013) for GRDDS with PEO alone. 

*Scanning electron microscopy (SEM)*

**Figure 7:**
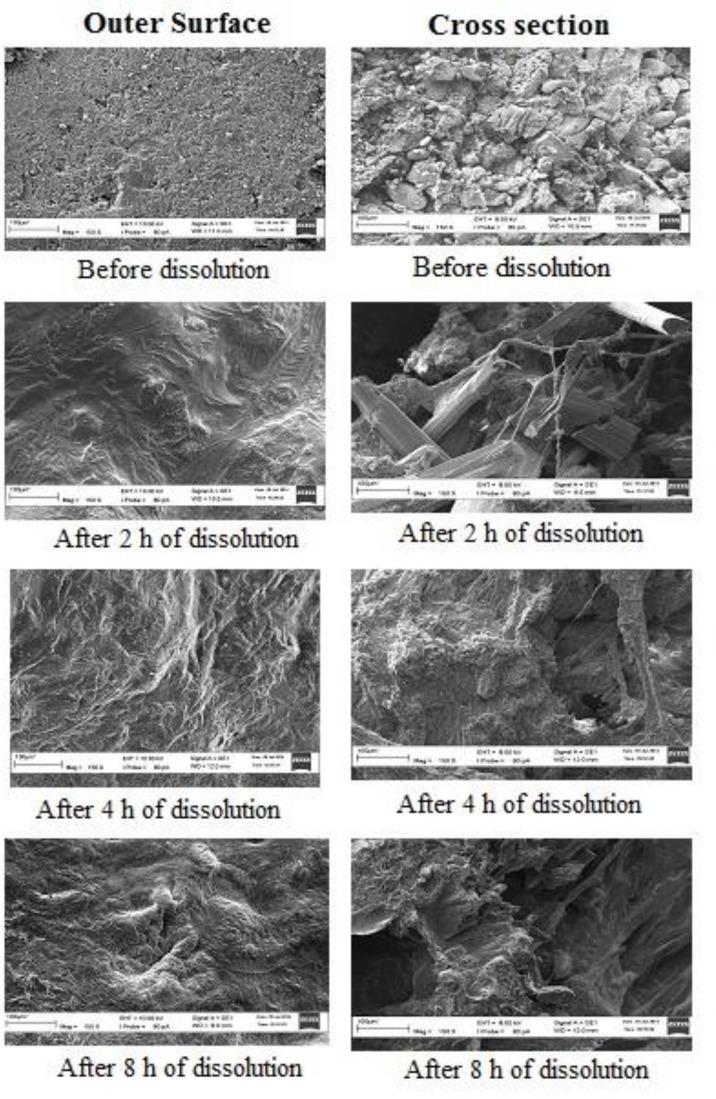
SEM pictures (150X magnification) of outer surface and inner surface (cross section) of tablets before and after 2 h, 4 h, and 8 h of dissolution study

SEM figures (Figure 7) depicted non-porous nature of tablet’s outer surface and little bit porous structure of its inner surface before dissolution study. However, for both the cases, the powder mass looked dense and compact. After dissolution of 2 h, the surfaces began to swell and become porous which allowed the drug to diffuse out in the dissolution medium. However, this swelling was more prominent in the outer surface as compared to the inner surface which resulted in the initial higher rate of drug dissolution. After 4 h and 8 h, both the inner and outer surfaces equally turned into porous structure. However, swelling of polymers increased the effective path length for drug diffusion and hence, the drug release rate becomes constant, but lower than the initial 2 h rate. These SEM figures supported the theory of diffusion for the drug release mechanism from the developed gastro-retentive drug delivery system.

*Accelerated stability studies*

**Figure 8:**
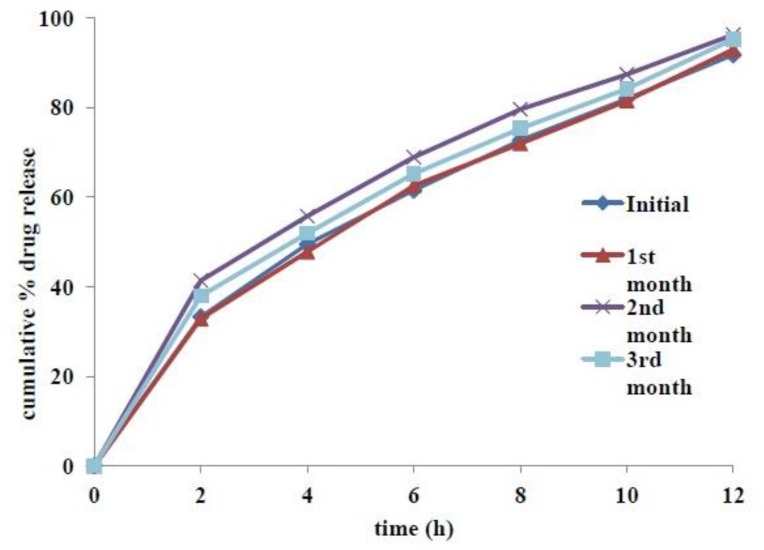
Dissolution profiles of tablets kept at accelerated stability conditions at initial, 1st, 2nd and 3rd months

Tablets from the optimized formulation were found to be stable, both physically and chemically, for a period of 3 months at accelerated stability conditions (40˚C and 75% RH). Physicochemical parameters, including appearance, hardness, friability, % moisture content, assay and floating lag time were not altered significantly. Results of assay and other evaluation criteria at periodic time points of stability studies are summarized in Table 9. Slight increase of tablet hardness in the second month stability samples might be related to their low moisture content. Increase Comparative dissolution profiles at various time points of accelerated stability studies are given in Figure 8. The calculated f2 value of first, second and third month’s stability samples compared with initial sample were found to be 90, 55 and 69 respectively, which indicated insignificant difference among the release profiles.

## Conclusion

Sustained-release floating tablets of metformin HCl was developed. Tablets with the ability to float *in-vitro* due to the formation of CO_2_ and the entrapment of generated CO_2_ by swelled polymeric matrix were fabricated. *In-vitro* floating behavior and drug release profile of developed tablets were influenced by the amount of polymer matrix (amount of HPMC and PEO), effervescent agent (sodium bicarbontate), and swelling enhancer (SSG). The proposed tablet composition was optimized by response surface methodology (RSM). Optimized tablets were able to float in less than 4 min, remained floating for more than 24 h and sustained the drug release for 12 h. Tablets from optimized formulation were stable both physically and chemically when stored for 3 months at accelerated stability conditions. Considering the promising *in-vitro* results of this study, it can be concluded that developed gastro retentive floating tablets of metformin HCl might be a better alternative than the conventional antidiabetic medications of the drug available in the market. However, clinical experiments on human should be conducted with the optimized formulation in order to correlate *in-vivo* performance with its* in-vitro* behavior. Additionally, combination of HPMC and PEO can be exploited in the future as swelling polymer to efficiently increase the residence time of drug in the stomach for sustained-release floating drug delivery system. 
